# Soil Nutrient Content Influences the Abundance of Soil Microbes but Not Plant Biomass at the Small-Scale

**DOI:** 10.1371/journal.pone.0091998

**Published:** 2014-03-17

**Authors:** Kadri Koorem, Antonio Gazol, Maarja Öpik, Mari Moora, Ülle Saks, Annika Uibopuu, Virve Sõber, Martin Zobel

**Affiliations:** 1 Department of Botany, Institute of Ecology and Earth Sciences, University of Tartu, Tartu, Estonia; 2 Department of Zoology, Institute of Ecology and Earth Sciences, University of Tartu, Tartu, Estonia; USDA-ARS, United States of America

## Abstract

Small-scale heterogeneity of abiotic and biotic factors is expected to play a crucial role in species coexistence. It is known that plants are able to concentrate their root biomass into areas with high nutrient content and also acquire nutrients via symbiotic microorganisms such as arbuscular mycorrhizal (AM) fungi. At the same time, little is known about the small-scale distribution of soil nutrients, microbes and plant biomass occurring in the same area. We examined small-scale temporal and spatial variation as well as covariation of soil nutrients, microbial biomass (using soil fatty acid biomarker content) and above- and belowground biomass of herbaceous plants in a natural herb-rich boreonemoral spruce forest. The abundance of AM fungi and bacteria decreased during the plant growing season while soil nutrient content rather increased. The abundance of all microbes studied also varied in space and was affected by soil nutrient content. In particular, the abundance of AM fungi was negatively related to soil phosphorus and positively influenced by soil nitrogen content. Neither shoot nor root biomass of herbaceous plants showed any significant relationship with variation in soil nutrient content or the abundance of soil microbes. Our study suggests that plants can compensate for low soil phosphorus concentration via interactions with soil microbes, most probably due to a more efficient symbiosis with AM fungi. This compensation results in relatively constant plant biomass despite variation in soil phosphorous content and in the abundance of AM fungi. Hence, it is crucial to consider both soil nutrient content and the abundance of soil microbes when exploring the mechanisms driving vegetation patterns.

## Introduction

Resource heterogeneity in space and time is a ubiquitous phenomenon in natural ecosystems and plays a fundamental role in determining plant community dynamics [1], [2]. Small-scale resource heterogeneity can be especially important in determining plant productivity since plants can concentrate their root biomass in areas of high nutrient content [3]–[5]. However, plant growth can also be strongly influenced by soil microorganisms [6]. Symbiotic microorganisms, such as mycorrhizal fungi, contribute to plant nutrient uptake, thereby affecting plant growth and biomass allocation [7]. Similarly, bacteria play an important role in increasing nutrient availability for plants [6]. Despite increasing recognition of the roles played by soil nutrients and especially soil microbes in determining vegetation patterns, information about how soil nutrients, microbes and plant biomass covary in natural ecosystems remains limited.

The spatial distribution of soil microbes is heterogeneous at various scales [8], [9] and can be closely related to soil fertility [7], [10]–[12]. Less is known about how temporal variation in soil nutrient content [1] influences soil microbial communities, though the abundance of fungi and bacteria has been shown to display significant temporal variation, with maximum abundance in spring and autumn [13]. Indeed, the abundance of soil microbes is expected to be high in spring and autumn when nutrient availability is high and decline as the plant growth season proceeds and nutrient availability subsequently decreases [14]. However more evidence is needed to better understand these dynamics and the mechanisms driving them.

Studies have found significant small-scale patchiness in the abundance in AM fungi, ectomycorrhizal fungi, other fungi and bacteria [15]–[17] but knowledge about the simultaneous dynamics of different microbial groups remains limited. Both positive and negative associations in the abundance of co-occuring microbes have been reported: in saprotrophic fungi and bacteria [18] and AM fungi and bacteria [19], [20]. Positive interactions may be the result of one group providing substrate for the other [19], while negative interactions may indicate competition for nutrients [20]. A recent study demonstrated that high N:P ratio in soil can provide an advantage, in the form of plant assimilated carbon, to AM fungi, in competition with non-mycorrhizal fungi that also inhabit plant roots [21].

Mycorrhizal fungi contribute to plant nutrient acquisition from the soil [7] and their abundance has been found to decrease with increasing nutrient availability [10], [11], [22]. Hence, mycorrhizal fungi and other soil microbes are expected to play an important role in determining plant productivity in ecosystems with low soil nutrients [6]. However, it is unknown whether the effect of soil microbes on plant productivity remains dependent on soil nutrient content at small scales. A recent experimental study did not find any change in the effect of AM fungi on plant productivity in conditions where local soil fertility varied [23], but that study did not consider the effect of microbes besides AM fungi.

We simultaneously measured the temporal and spatial variation of soil nutrient content, soil microbial biomass and plant biomass in a natural herb-rich boreonemoral spruce forest. We focused on a scale that corresponds to the approximate size of adult plant individuals in the understory (i.e. small scale, 15 cm^2^). The study site is characterized by heterogeneous light and soil conditions, patchy understory vegetation [24], and remarkably taxon rich AM fungal communities in the roots of herbaceous species [25], [26]. We ask:

i) Does soil nutrient content and microbial abundance vary during the first half of the growing season when plant growth is rapid?

ii) Are there negative associations in the small-scale spatial distribution of soil microbial groups?

iii) Is the spatial distribution of soil microbes and plant biomass related to soil nutrient content?

## Materials and Methods

### Study site and sampling design

The study was carried out in a boreonemoral forest near Koeru, Estonia (58°58′N; 26°03′E). The climate of the study site is transitional between maritime and continental. The mean annual precipitation in the area is 665 mm and the mean annual air temperature is 5.0 °C (−5.4 °C in January and 16.6 °C in July on average; Estonian Meteorological and Hydrological Institute, 2013). The study site is a 130 ha patch of *Hepatica* type forest on a calcareous cambisol (cf. [24]). The soil of the area is relatively uniform in terms of pH, ranging from 5.2–5.7, but exhibits some variation in nutrient content [27], [28]. The tree layer is dominated by *Picea abies* (L.) Karst. with individuals of *Fraxinus excelsior* L. and *Acer platanoides* L. also present. The shrub layer is dominated by *Corylus avellana* L. Common species in the herb layer are *Oxalis acetosella* L., *Hepatica nobilis* Schreb., *Paris quadrifolia* L. and *Viola mirabilis* L. The forest can be classified as relatively undisturbed old-growth forest, but clear cutting of approximately 1–2 ha patches has taken place repeatedly in parts of the study site. Detailed descriptions of the area are provided by Moora *et al.* [24] and Zobel *et al.* [27]. This study complies with the laws of Estonia in which no permits are required to carry out research on public land, unless specified otherwise in legislation. No regulation applies to the Koeru site and no protected species were sampled during the study.

Three 105×105 cm plots (A, B, C) were positioned at 5 m intervals in an area of homogenous forest in terms of vegetation cover and light conditions as estimated by eye. All plots were divided into 49 (15×15 cm) quadrats. During preliminary measurements conducted in summer 2007, five plant individuals of the five most common understory species (*Oxalis acetosella, Hepatica nobilis, Fragaria vesca* L., *Galeobdolon luteum* Huds., *Viola mirabilis*) were excavated to a depth of 15 cm, and the root systems of those plants measured with a ruler to determine the size of an average adult plant root system. Soil cores studied during the preliminary study harboured most of the roots in the upper 10 cm of the soil and the average size of plant roots systems was 15×15 cm (data not presented). The size of quadrats used in the main experiment therefore corresponds to the average size of the root system of an adult plant (the average of the species listed above), and is expected to harbour the majority of the biomass of herbaceous plant roots and microbes present in the rhizosphere. Plot A was used to address temporal variation, and plots A, B and C were used to address spatial variation in soil nutrient content and soil microbial activity at small scales. Plot A was sampled twice, at the beginning (26.05.2008) and in the middle (21.07.2008) of the growing season. The small spatial scale of the study meant that it was not possible to address temporal variation in the abundance of soil nutrients and microbes over a longer time period, as additional sampling would have necessitated considerable disturbance. Soil samples (50 g and 10 g) were collected from the soil surface (depth 10 cm) at both sampling times for soil chemical, and fatty acid analysis respectively. Soil samples collected at beginning of the plant growing season (in plot A only) were excavated with a soil corer (2 cm diameter and 10 cm deep) at the corner of each quadrat (n = 49) in order to minimize disturbance to the plots. The hole was filled with soil taken from nearby, to minimize sampling effects on soil conditions. Collected soil was mixed (to avoid any effect of vertical structure on measured parameters) and subjected to soil chemical and fatty acid analysis (see below). Soil samples collected in the middle of the growing season (July) were collected during destructive harvesting (see below). One core was taken from each quadrat; in plot A, the locations where the first samples were collected were avoided. The soil corer used during destructive harvesting had a diameter of 15 cm and a depth of 10 cm. All soil cores were transported to the laboratory where, after the removal of soil samples (50 and 10 grams as before) from all of the soil cores (n = 147), the shoots and roots of herbaceous plant species were separated carefully by hand from the samples from plots B and C (n = 98). Plant biomass was dried to a constant weight at 70 °C, and weighed. As the vegetation in plot A was disturbed by the sampling at the beginning of the growing season, biomass was not collected from that plot.

### Soil chemical and fatty acid analysis

Soil samples for chemical analyses were kept at −80°C prior to analysis, in order to prevent the loss of soil nutrients. Analysis of soil nutrient content followed International Organization for Standardization (ISO) protocols. Specifically, determination of soil ammonium (NH_4_) and nitrate (NO_3_) content (mg/kg) was based on ISO 14256–2: 2005. Organic carbon (C) and total nitrogen (N) content (%) were determined based on ISO 10694: 1995 and ISO 13878: 1998, respectively. As soil NH_4_, NO_3_ and C content were highly correlated with total N content, only the latter was used in further analysis. The Mehlich III procedure was used to determine soil phosphorus (P) and potassium (K) content (mg/kg): P was extracted by reaction with acetic acid and fluoride compounds; exchangeable K was extracted by the action of ammonium nitrate and nitric acid [29]. Chemical analyses were performed at the laboratory of the Agricultural Research Centre in Saku, Harjumaa, Estonia.

Ester-linked fatty acid (ELFA) content in soil samples was used as a proxy for the biomass of major microbial groups: AM fungi, other fungi and bacteria. Fatty acid profiling is widely used to measure microbial biomass and coarse microbial community composition [10], [30]–[35]. The abundance of soil microbes can also be estimated by quantifying their DNA by qPCR [35] but the advantage of fatty acid profiling is that it enables the biomass of different microbial groups to be determined simultaneously. Three grams of fresh soil (randomly taken from collected 10 g) was lyophilized immediately after separation and stored at room temperature. Phospholipids were converted to ester-linked fatty acids and extracted from the soil following the protocol of Schutter & Dick [36], which methylates only ester-linked and not free fatty acids. Briefly, 15 ml of 0.2 M KOH in methanol was added to 2 g of dry soil, mixed and incubated with shaking at 200 rpm at 37°C for 1 h. The sample was neutralized with 3 ml of 1 M acetic acid. Fatty acid methyl esters were extracted by adding 10 ml of hexane, the sample was mixed by vortexing and left to settle into two layers. The upper hexane layer was transferred to a clean tube and evaporated under a vacuum in a Jouan RC1022 Vacuum Concentrator. Fatty acids were re-suspended in 100 μl hexane and transferred to a GC vial for analysis using gas chromatography.

The ester-linked fatty acid peak areas were normalized against the internal standard and expressed as μg per g of dry soil. Fatty acid nomenclature follows Ranneklev & Bååth [37]. Ester-linked fatty acid 16:1ω5c was used as a biomarker for AM fungi. This marker is widely used as an indicator for AM fungi [30], [32], though the different fatty acid fractions of cells may be of different indicative value for AM fungi. Namely, NLFA (neutral lipid fatty acids, storage lipids) 16:1ω5c occurs only in AM fungi, while PLFA (phospholipid fatty acids, structural lipids in cell membranes) 16:1ω5c can also occur in some Gram-negative bacteria [38], [39]. NLFA is therefore proposed as a more suitable indicator of AM fungi in samples with potentially high-bacterial content, such as soils [39], [40]. However, ELFA 16:1ω5c has been shown to correlate very well with AM fungal inoculation in an experiment containing both AM fungi and microbes from the AM fungal inocula, where ELFA 16:1ω5c was absent in microbial wash-treated, but AM fungal-free soils [41]. Therefore, ELFA measurements used in this paper represent whole-cell fatty acids, i.e. they contain both the PLFA and NLFA fraction. Fatty acid 18:2ω6,9 was used as a biomarker for fungi other than AM fungi, as it is a dominant fatty acid in most fungi [31] but is only found at very low levels in AM fungi [30]. We calculated the ratio between the levels of fatty acids 16:1ω5c and 18:2ω6,9 (hereafter AM fungi: other fungi ratio) to estimate the relative abundance of AM fungi in the soil fungal community. Fatty acids i15:0, a15:0, 15:0, i16:0, 16:1ω9c, br17:0, 10Me16:0, i17:0, a17:0, 17:0, cy17:0, br18:0, 10Me17:0, 18:1ω7c, 10Me18:0, cy19:0 ω 9, cy19:0 ω 7 were used as bacterial biomarkers [31], [42].

### Statistical analyses

Linear Mixed-Effect Models (LMM) [43] were used to address temporal differences in soil nutrient content (N, P, K) and soil microbial biomass (fatty acid biomarkers for AM fungi, other fungi, AM fungi: other fungi ratio, bacteria). The abundance of other fungi and bacteria as well as the AM fungi: other fungi ratio was log transformed prior to analysis to meet the assumptions of parametric tests. For each variable, a model using time (sampling time: May or July) as a fixed factor and quadrat identity as a random factor were fitted. The homogeneity assumption for model residuals was evaluated graphically [44]. When any sign of heterogeneity was detected in the residuals, it was tested if keeping the variance fixed between the two time periods resulted in homogeneity for the residuals (i.e. including a fixed variance structure in the model; [44]). Models were fitted using Restricted Maximum Likelihood (REML) in order to compare models with different variance structures [44].

Moran's I was used to determine the presence of spatial autocorrelation in the distribution of soil microbes. Moran's I calculates the degree of correlation between observations as a function of the spatial distance separating them [45]. A correlogram is a graphical representation of the Moran's I statistic against distance classes, and provides information about the spatial autocorrelation of a variable and the size of patches. We constructed a spatial correlogram for each group of soil microbes (AM fungi, other fungi and bacteria) in each plot (A, B and C). Moran's I was evaluated at different distance classes (0 to 50 cm) and its significance was assessed by performing 9999 unrestricted permutations. Since the quadrats were distributed regularly, all correlograms were constructed using distance classes with increments of 0.075 m up to a maximum distance of 0.75 m. The first distance class (0.000–0.075) was discarded since it did not contain any pairs of quadrats.

The Modified t-Test for correlation [46] was used to study pair-wise relationships between the abundance of soil microbes: AM fungi, other fungi and bacteria in each plot (A, B and C). This method evaluates the correlation between two spatial patterns using the Pearson's product-moment correlation, but corrects the significance of the correlation for the presence of spatial autocorrelation.

Generalized least square models (GLS) [43] were used to determine which factors best explain variations in soil microbial abundance (in plots A, B, C) and both above- and belowground biomass of herbaceous plants (in plots B, C). The set of predictor variables were different depending on the response variable considered. Specifically, in the models of soil microbial abundance (AM fungi, other fungi, bacteria and AM fungi: other fungi ratio), the influence of plot identity (A, B and C) and soil nutrients (N, P, K) were considered. In the models of above- and belowground biomass of herbaceous plants, the influence of plot identity, soil nutrients and microbial abundance were considered. In order to select the most parsimonious model for each variable, the protocol proposed by Zuur *et al.* [44] was followed. First, a model which included the full set of covariates as potential predictors was fitted for each variable. After that, the homogeneity of the residuals of the model was evaluated graphically. In addition, due to the nested structure of the sampling design, within plot spatial autocorrelation of residuals was studied. When spatial autocorrelation was present, the inclusion of correlation structures of different form were evaluated [47]. Finally, models were fitted with Maximum Likelihood (ML) and the best set of predictor variables was selected using Akaike's Information Criterion (AIC). If several models showed similar AIC values (difference lower than two), the most parsimonious one, i.e. the one with fewer fixed components, was chosen [44]. The final model was then refitted using REML to obtain estimates of factor effects and their significance.

LMM, GLS and modified t-Tests and correlograms were performed in the R environment [48], using the packages nlme [49], SpatialPack [50] and ncf [51]. Maps of the spatial patterns of AM fungal abundance and soil P content were created using ArcGIS 9.3 software (ESRI Redlands, NY, USA). For each variable, the “geostatistical analyst” extension of ArcGIS 9.3 was used to generate semivariograms of density and create kriging maps.

## Results

### Temporal variation

**Figure 1:**
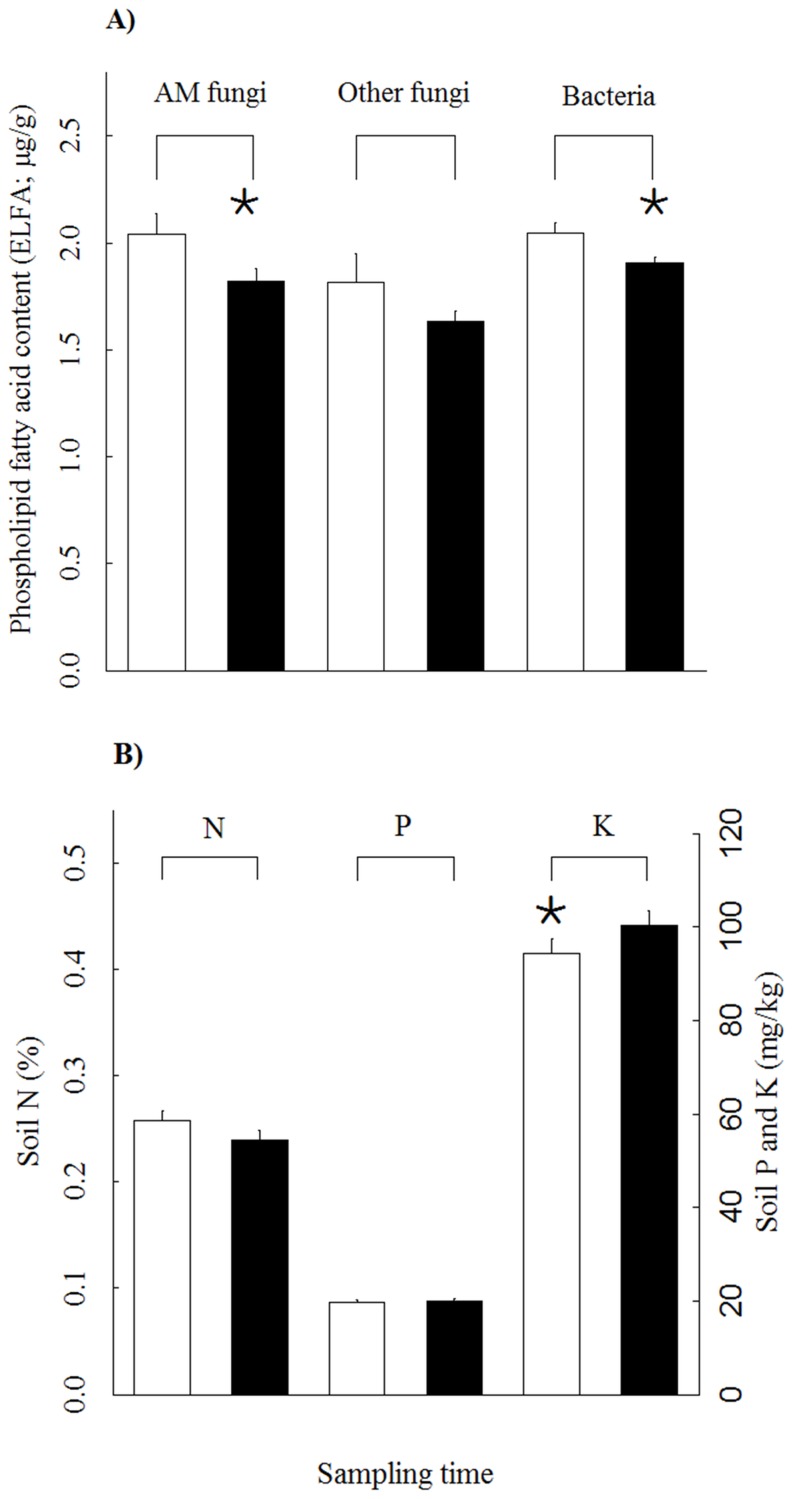
The abundance of soil microbes (A) and nutrients (B) in May (open bars) and July (closed bars). The abundance of arbuscular mycorrhizal (AM) fungi, other fungi and bacteria was estimated as the content of respective ester-linked fatty acid (y-axis on graph A, see Methods for details). Soil N concentration was measured in % (left y-axis of graph B) whereas P and K content were measured as mg/kg (right y-axis of graph B). Significant differences (Linear Mixed-Effect Models; p<0.05) in the measured parameters over time are marked with *.

After the removal of outliers, data from 46 quadrats from plot A was subjected to analysis of temporal variation in soil nutrient content and soil microbial abundance. The linear mixed-effect models (LMMs) revealed significant temporal variation in the abundance of arbuscular mycorrhizal (AM) fungi, bacteria and soil K content, but not in the other variables (Table 1, Figure 1). Means of the measured variables are given in Table S1. The abundance of AM fungal and bacterial fatty acid biomarkers in the soil was higher in May than in July. Soil K content was lower at the beginning of the growing season. The model fitted to explain temporal variation in the abundance of AM fungi included a fixed variance structure as it substantially improved the model fit.

**Table 1:** Summary statistics of the Linear Mixed-Effect Models analysis fitted to study the influence of sampling time (May or July) on soil nutrient content and ester-linked fatty acid (ELFA) biomarkers of arbuscular mycorrhizal (AM) fungi, other fungi and bacteria measured from plot A.

Variable	Num DF	Den DF	F	P
Nitrogen (%)	1	45	3.43	0.07
Phosphorus (mg/kg)	1	45	0.15	0.70
Potassium (mg/kg)	1	45	5.20	0.03
AM fungi (ELFA 16:1ω5c μg g−1 soil)	1	45	4.87	0.03
Other fungi (ELFA 18:2ω6,9 μg g−1 soil)	1	45	1.16	0.29
AM fungi: other fungi	1	45	0.79	0.38
Bacteria (ELFA μg g−1 soil)	1	45	6.53	0.01

The degrees of freedom of the numerator (Num DF) and denominator (Den DF), F statistic (F) and associated probability (P) for the sampling time (i.e. fixed factor) are presented.

### Spatial variation

After the outliers were removed 47, 46 and 44 quadrats from plots A, B and C respectively were subjected to analysis to test the spatial variation of soil nutrient content and the abundance of soil microbes. Limited evidence was found for the presence of spatial autocorrelation in the abundance of soil microbes studied in each plot, since only the abundance of bacteria showed significant autocorrelation (Figure S1). The abundance of AM fungi and soil bacteria tended to show positive spatial autocorrelation up to a distance of 20 cm, but this trend was significant only in the case of bacteria for plots A and C (Figure S1). The Modified t-Test for correlation revealed significant correlations between the abundance of AM fungi and other fungi, AM fungi and bacteria and between bacteria and other fungi (Table 2).

**Table 2:** Results of the modified t-Test for correlation performed to study the relationship between the abundance of arbuscular mycorrhizal (AM) fungi, other fungi and bacteria in the three plots (A, B, C).

		Other fungi	Bacteria
AM fungi	Plot A	0.44**	0.53**
	Plot B	0.54**	0.36*
	Plot C	0.81**	0.56**
Other fungi	Plot A	–	0.41**
	Plot B	–	0.54**
	Plot C	–	0.55**

For each plot, the Pearson's moment correlation coefficient is presented, accompanied by its significance corrected by the presence of spatial autocorrelation. * P<0.05; ** P<0.01.

**Figure 2:**
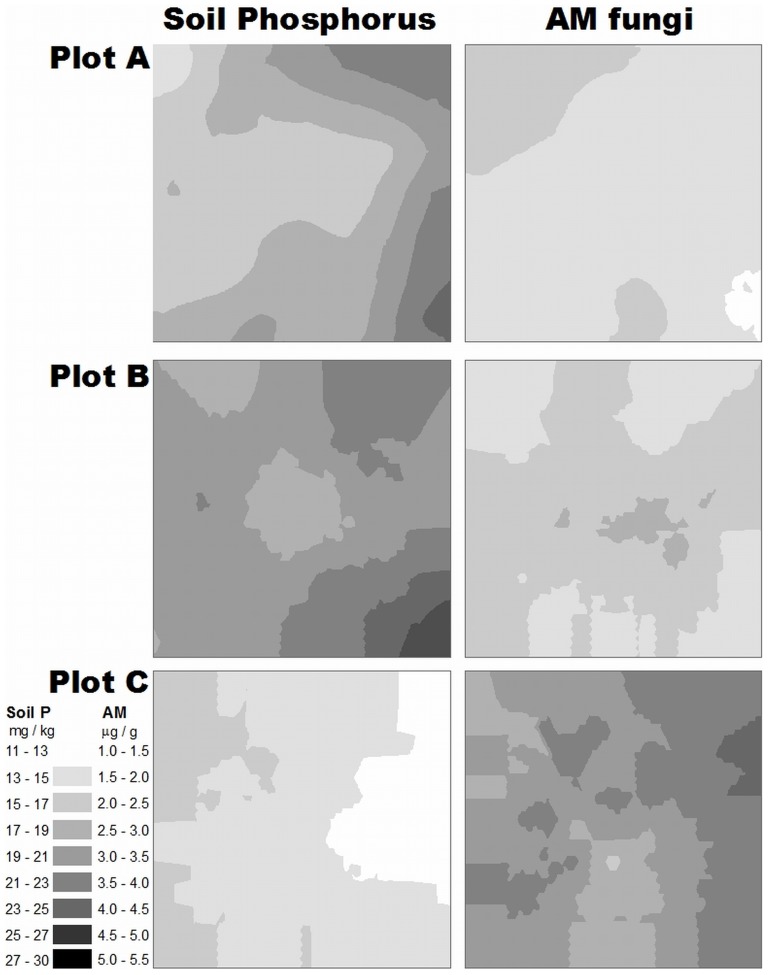
Spatial pattern of soil phosphorus content and abundance of arbuscular mycorrhizal fungi (ester-linked fatty acid 16:1ω5c in soil) in plots A, B and C. Maps were created by kriging, using data from soil samples collected from 15×15 cm quadrats in each 105×105 cm plot.

Generalized least square models revealed that plot identity was an important predictor of the abundance of AM fungi (Table 3); the mean abundance was significantly lower in plots A and B compared to plot C (Table 4). In addition, the abundance of AM fungi was significantly positively related to the content of soil N and negatively affected by soil P content (Table 3, Figure 2). Plot identity also played a significant role in predicting the abundance of other fungi (Table 3); the mean abundance was considerably higher in plot C compared to plots A and B (Table 4). In addition, the abundance of other fungi was significantly positively influenced by soil K (Table 3). The AM fungi: other fungi ratio was also predicted by plot identity (Table 3); the mean AM fungi: other fungi ratio differed significantly between all of the plots, being lowest in plot A and highest in plot C (Table 4). In addition, the AM fungi: other fungi ratio was negatively associated with soil P content (Table 3). The abundance of bacteria was the only variable that was best explained by a model where a spatial correlation structure was included, indicating small-scale autocorrelation in bacterial biomass. The abundance of bacteria was explained by plot identity (Table 3), as the mean differed significantly between all plots, being lowest in plot A and highest in plot C (Table 4). In addition, the abundance of bacteria was significantly positively influenced by soil K content (Table 3).

**Table 3:** Results of the generalized least square models performed to study the influence of environmental conditions on soil microbes.

	Plot B	Plot C	Nitrogen	Phosphorus	Potassium	AIC weight	R^2^
AM fungi	0.206*	1.051**	2.874*	−0.074**	–	0.36	0.57
Other fungi	0.014	0.627**	–	–	0.003*	0.71	0.37
AM: other fungi	0.151**	0.166**	–	-0.024**	–	0.23	0.23
Bacteria	1.612**	3.874**	–	–	0.017*	0.36	0.36

For each soil microbial group, the coefficient associated with the explanatory variable is presented. In addition, the AIC weight of the regression model (i.e. its importance as compared to other models containing a different subset of explanatory variables) and the R^2^ are shown. * P<0.05; ** P<0.01.

**Table 4:** The mean, standard deviation (SD) and the range of the soil nutrient content, ester-linked fatty acid (ELFA) biomarkers of AM fungi, other fungi, bacteria and vegetation characteristics measured at 1 m^2^ scale.

Variable	Plot A		Plot B		Plot C	
	Mean (±SD)	Range	Mean (±SD)	Range	Mean (±SD)	range
Nitrogen (%)	0.24±0.07^a^	0.15–0.43	0.27±0.05^b^	0.15–0.41	0.24±0.03^a^	0.18–0.30
Phosphorus (mg/kg)	20.11±3.02^a^	13.00–28.00	20.63±2.63^a^	15.00–30.00	14.16±1.64^b^	12.00–18.00
Potassium (mg/kg)	100.43±21.07^a^	57.00–159.00	92.80±22.58^a^	54.00–159.00	68.59±10.56^b^	47.00–93.00
AM fungi (ELFA 16:1ω5c μg g-1 soil)	1.83±0.42^a^	1.16–2.95	2.09±0.52^a^	1.22–3.07	3.32±0.81^b^	1.56–4.98
Other fungi (ELFA 18:2ω6,9 μg g-1 soil)	1.65±0.30^a^	1.02–2.36	1.64±0.35^a^	1.02–2.67	2.18±0.39^b^	1.49–2.88
AM fungi: other fungi	1.13±0.24^a^	0.59–1.67	1.29±0.24^b^	0.68–1.72	1.52±0.22^c^	1.04–1.93
Bacteria (ELFA μg g-1 soil)	19.14±1.61^a^	16.08–22.38	20.58±1.75^b^	16.20–25.50	22.54±2.30^c^	15.64–26.85
Root biomass of woody species (g)	–	–	10.30±6.76	2.30–37.00	8.92±5.53	1.80–28.10
Shoot biomass of herbaceous species (g)	–	–	0.81±0.42	0.16–2.06	0.79±0.36	0.04–1.53
Root biomass of herbaceous species (g)	–	–	0.57±0.63	0.03–3.07	0.64±0.53	0.03–1.88

Due to destructive sampling, vegetation characteristics were only measured in plots B and C (see explanation in Methods). Different letters (when present) mark a significant difference among means according to Tukey HSD test (p<0.05).

Of all the variables considered, the AM fungi: other fungi ratio was the only one with a significant and positive effect on the shoot biomass of herbaceous plants (F = 5.29; P = 0.02). However, the mean shoot biomass of herbaceous plants did not differ between plots (Table 4). None of the studied variables explained the root biomass of herbaceous plants, which also did not vary significantly between plots (Table 4).

## Discussion

The small-scale abundance of soil microbes varied through time in the studied boreonemoral herb-rich spruce forest. We recorded a lower abundance of soil microbes in the middle compared to the beginning of the growing season, whereas no decrease in soil nutrient content occurred. Contrary to our expectations, the small-scale spatial abundance of different soil microbes, including bacteria, arbuscular mycorrhizal (AM) fungi and other fungi, was positively related, suggesting little or no importance of resource competition among these groups in our study system. However, the abundance of soil microbes was significantly influenced by soil nutrient content. In particular, the abundance of AM fungi was strongly negatively related to soil P content. Plant biomass on the other hand showed little variation. Based on these results, we suggest that plants potentially compensate for low soil P content with alternative resource acquisition mechanisms, for example via symbiosis with AM fungi, and therefore achieve equal biomass production despite variations in soil nutrient content and the abundance of soil microbes. Soil microbes (especially plant symbionts such as AM fungi) thus play a fundamental role in the relationship between soil nutrients and plant productivity.

The abundance of soil microbes (especially AM fungi and bacteria) was lower in the middle of the plant growing season, which matches the findings of previous studies that have addressed the temporal variation of soil microbes at larger and smaller spatial scales [13], [52]. Active plant growth during the first half of the growing season did not bring about a decrease in soil nutrient content. Thus, the importance of soil nutrients in determining temporal changes in the abundance of soil microbes seem to be minor [52]. Some previous studies have reported a contrasting pattern of AM fungal abundance during the plant growing season: an initial increase with a peak in the summer, followed by a decrease in the autumn [53], [54]. A potential explanation for this inconsistent variation of AM fungal abundance in soil can be the particular weather conditions of the sampling year, as soil AM fungal hyphal density is considerably affected by temperature and precipitation [54], [55]. Thus, data from long-term measurements with multiple sampling times during the growing season is needed in order to draw solid conclusions about the dynamics of soil microbes.

We found a strong positive association between the abundance of bacteria, AM fungi and other fungi, suggesting a limited role of resource competition among these microbial groups in a natural ecosystem. Several previous experimental studies have reported negative associations between the abundance of ectomycorrhizal fungi, AM fungi and bacteria [20], [56], as well as between the abundance of ectomycorrhizal and saprotrophic fungi [57], and AM fungi and other fungi [21], [58]. On the other hand, studies conducted in natural conditions have demonstrated similar biomass dynamics of fungi other than AM fungi and bacteria during both the growing season [13] and over decades [59]. These contrasting results highlight the importance of studies addressing the pattern of soil microbes in natural environments. The strong positive relationship between the abundance of bacteria, AM fungi and other fungi found in this study suggests that the use of slightly different resources by soil microbes promotes their coexistence. Indeed, AM fungi, which are expected not to be highly saprotrophic themselves, have been shown to promote the activity of saprotrophic microbes [60]. In addition, fungal hyphae and spores can function as substrates for bacteria [19], [61], and carbon released from dead bacteria can promote the growth of saprotrophic fungi [32]. Also, living roots excrete carbon compounds [62], which can be consumed by saprotrophic soil microorganisms [13]. In addition, AM fungi as well as ectomycorrhizal fungi have been shown to increase their growth in the presence of root exudates [63], [64].

The abundance of all groups of soil microorganisms tested depended on the sampling location (plot identity), which confirms the small-scale spatial heterogeneity of soil microbes previously demonstrated [12]. In addition, soil nutrients played an important role in determining the abundance of soil microorganisms. The negative effect of soil P content on the abundance of AM fungi has also been reported in earlier field fertilization experiments in grasslands and forests [65], [66]. As AM fungi can improve the acquisition of soil nutrients, especially P, by plants [7], [67], they can play an important role when soil nutrient content is low. When soil nutrient levels increase, the cost to a plant of supporting AM fungi may outweigh the benefits, and can result in a decrease of AM fungal colonization of plant roots [7]. However, soil AM fungal fatty acid content has also been reported to be positively or insignificantly related to soil P content [68], [69]. We recorded a positive effect of soil N on the abundance of AM fungi, a pattern that has also been registered at a large scale [35] and can be explained by the high N demand of AM fungi [70]. In addition, AM fungal communities have been shown to be highly influenced by soil pH [71]. However, Dumbrell *et al.* [71] focused on sites with high variation in soil pH (ranging from 4.5–8), whereas our study area is characterised by fairly uniform pH values [27], [28]. Therefore, the role of soil nutrients in determining the abundance of AM fungi in current study site could be expected to be more pronounced, while the effect of small-scale variation in soil pH remains to be fully considered in future studies.

Saprotrophic and ectomycorrhizal fungi as well as bacteria were positively influenced by soil K content. A recent study demonstrated an increase in bacterial biomass, and growth of extramatrical mycelia of ectomycorrhizal fungi in response to K addition in peatland soils with severe K deficiency [72]. Our results suggest that K also has an important role to play in determining the abundance of soil microbes in soils where K content is not extremely low. On the other hand, the observed positive relationship can also reflect increased weathering and K influx resulting from active growth of bacteria and saprotrophic fungi [73], [74].

Plants have been shown to concentrate their biomass into patches with high nutrient content [3]–[5], but in the current study, conducted in an old and stable ecosystem, we found no direct effect of nutrients on plant biomass. One explanation for the absence of the expected pattern is that plants are continuously foraging, while the pattern of biomass measured in studies such as this one reflects dynamics that have operated over numerous years. Greenhouse experiments have shown that nutrient poor patches contain higher plant biomass when next to nutrient rich patches [4], [75] but those experiments were short-term. In the longer term, there will be natural turnover of nutrient rich patches (both temporal and spatial), and active foraging can therefore result in relatively uniform plant biomass. Secondly, the natural variation in nutrient content could be too low to generate distinct patterns. Indeed, understory plant biomass in this study area has not shown changes in response to induced variation in soil nutrient content either [23]. Thirdly, the effect of soil nutrients can be mitigated by the influence of soil microbes such as AM fungi. AM fungi seemed to be the only group of soil microbes to affect understory plant growth, as the relative increase of AM fungi compared to other fungi resulted in increased plant shoot biomass. As AM fungi can greatly improve plant P acquisition [7], [67], the presence of AM fungi can be more important for plant growth when soil P content is low and less beneficial for plants when soil nutrient content is high. In accordance with this, a decrease of AM fungal hyphal density in soil and AM colonization in plant roots has been reported in response to increased P availability [22], [76]; whereas the nutrient content of plant tissues and plant biomass did not exhibit any change [22], [68]. Our study, based on data from a natural environment, demonstrates stable plant biomass in conditions of low abundance of AM fungi and high P content and *vice versa.* We support the suggestion of Beauregard *et al.* [68] that plants can compensate for low available nutrient concentration with more efficient symbiosis with soil microbes. Hence, it is crucial to consider both the soil nutrient content and the abundance of soil microbes when exploring the mechanisms driving vegetation patterns.

## Supporting Information

Figure S1 Spatial correlograms of microbes. Spatial correlograms of arbuscular mycorrhizal fungi (a–c), other fungi (d–f), and bacteria (g–i) in plot A (a, d, g), B (b, e, h) and C (c, f, i). Distances with significant spatial autocorrelation are marked with solid circles.
(PDF)

Table S1 Temporal variation in the abundance of soil nutrients and microbes. Summary statistics of the soil nutrient content and ester-linked fatty acid (ELFA) biomarkers of arbuscular mycorrhizal (AM) fungi, other fungi and bacteria measured from plot A at the beginning (May) and in the middle (July) of the growing season. The mean, standard deviation (SD) and the range are presented for each variable.
(PDF)
