# Supplementary figures and images for: Soil Nutrient Content Influences the Abundance of Soil Microbes but Not Plant Biomass at the Small-Scale
Source: PLoS One. 2014 Mar 17;9(3):e91998. doi: 10.1371/journal.pone.0091998 (PMC3956881; doi:10.1371/journal.pone.0091998)

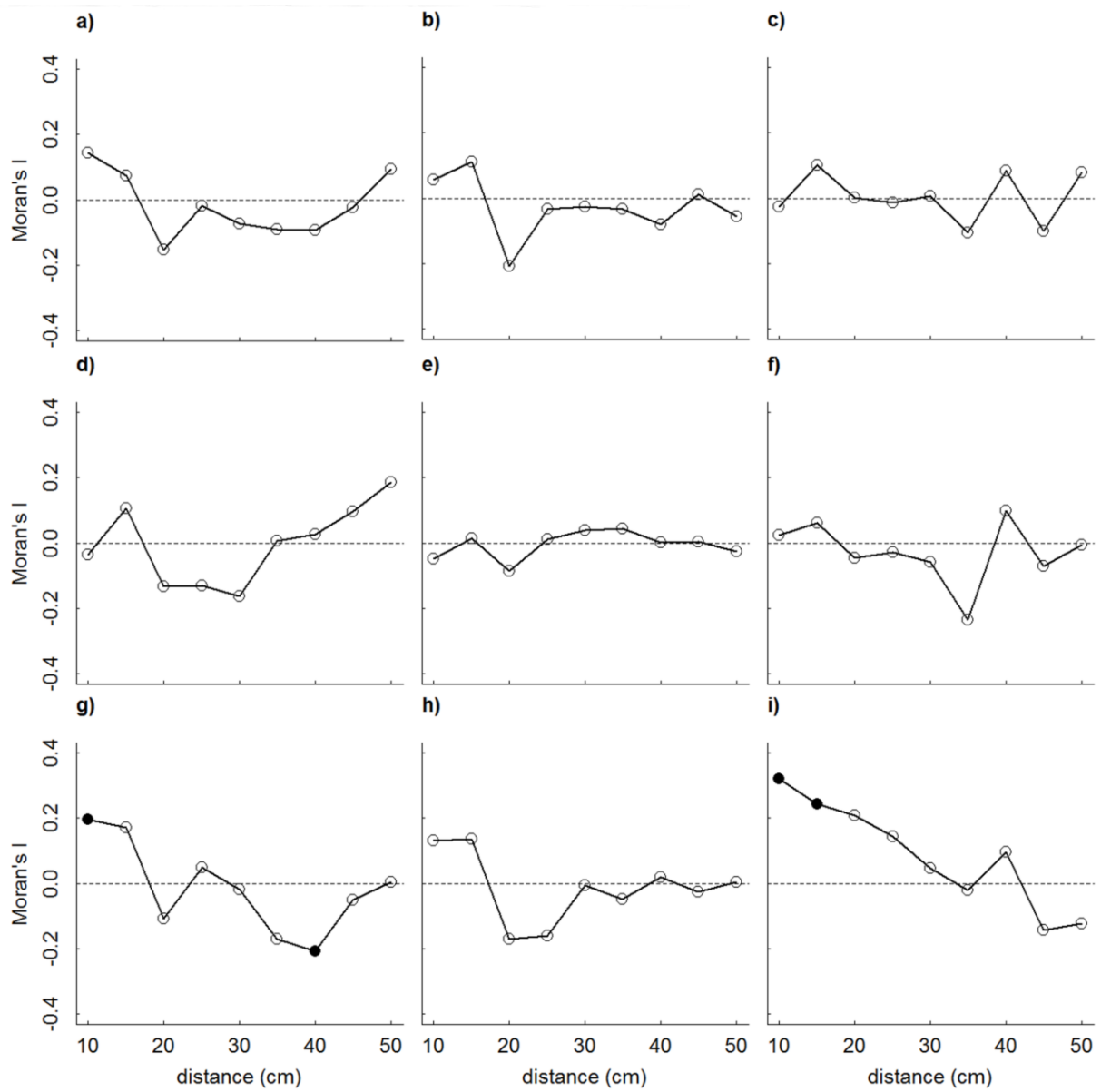

Supplement: Figure S1 — Spatial correlograms of microbes. Spatial correlograms of arbuscular mycorrhizal fungi (a–c), other fungi (d–f), and bacteria (g–i) in plot A (a, d, g), B (b, e, h) and C (c, f, i). Distances with significant spatial autocorrelation are marked with solid circles. (PDF) [file pone.0091998.s001.pdf]
