# Supplementary material for: Soil Nutrient Content Influences the Abundance of Soil Microbes but Not Plant Biomass at the Small-Scale
Source: PLoS One. 2014 Mar 17;9(3):e91998. doi: 10.1371/journal.pone.0091998 (PMC3956881; doi:10.1371/journal.pone.0091998)
Supplement: Table S1 — Temporal variation in the abundance of soil nutrients and microbes. Summary statistics of the soil nutrient content and ester-linked fatty acid (ELFA) biomarkers of arbuscular mycorrhizal (AM) fungi, other fungi and bacteria measured from plot A at the beginning (May) and in the middle (July) of the growing season. The mean, standard deviation (SD) and the range are presented for each variable. (PDF) [file pone.0091998.s002.pdf]

Variable	May		July	
	Mean (±SD)	Range	Mean (±SD)	Range
Nitrogen (%)	0.26 ± 0.06	0.13 – 0.42	0.24 ± 0.07	0.15 – 0.43
Phosphorus (mg/kg)	19.82 ± 2.69	15.00 – 27.00	20.06 ± 2.99	13.00 – 28.00
Potassium (mg/kg)	96.98 ± 24.85	55.00 – 175.00	100.22 ± 20.74	57.00 – 159.00
AM fungi (ELFA 16:1ω5c µg g-1 soil)	2.05 ± 0.65	1.15 – 4.26	1.83 ± 0.42	1.16 – 2.95
Other fungi (ELFA 18:2ω6,9 µg g-1 soil)	1.82 ± 0.88	1.05 – 7.03	1.68 ± 0.39	1.02 – 3.40
AM fungi : other fungi	1.18 ±0.30	0.38 – 2.08	1.13 ± 0.24	0.59 – 1.67
Bacteria (µg ELFA g-1 soil)	20.44 ± 3.42	15.06 – 37.79	19.28 ± 1.85	16.08 ± 25.67
